# The identification and genetic characteristics of *Quang Binh virus* from field-captured *Culex tritaeniorhynchus* (Diptera: Culicidae) from Guizhou Province, China

**DOI:** 10.1186/s13071-023-05938-3

**Published:** 2023-09-07

**Authors:** Xiaomin Tang, Rongting Li, Yanfei Qi, Weiyi Li, Zhihao Liu, Jiahong Wu

**Affiliations:** 1 Characteristic Key Laboratory of Modern Pathogen Biology, School of Basic Medicine, Guizhou Medical University, Guiyang, 550025 China; 2 Department of Human Parasitology, School of Basic Medicine, Guizhou Medical University, Guiyang, 550025 China; 3 School of Public Health, Guizhou Medical University, Guiyang, 550025 China; 4 College of Osteopathic Medicine, Duquesne University, Pittsburgh, PA 15282 USA; 5 College of Osteopathic Medicine, California Health Sciences University, Clovis, CA 93611 USA

**Keywords:** Mosquito, Flavivirus, Quang Binh virus, Insect-specific flaviviruses, *Culex tritaeniorhynchus*, Genome, Guizhou Province

## Abstract

**Background:**

Mosquitoes carry a variety of viruses that can cause disease in humans, animals and livestock. Surveys for viruses carried by wild mosquitoes can significantly contribute to surveillance efforts and early detection systems. In addition to mosquito-borne viruses, mosquitoes harbor many insect-specific viruses (ISVs). Quang Binh virus (QBV) is one such example, categorized as an ISV within the *Flavivirus* genus (family *Flaviviridae*). QBV has been specifically documented in Vietnam and China, with reports limited to several mosquito species.

**Methods:**

The homogenate obtained from female mosquitoes was cultured on C6/36 (*Aedes albopictus*) and BHK-21 (baby hamster kidney) cell lines. Positive cultures were identified by reverse transcription-polymerase chain reaction (RT‒PCR) with taxon- or species-specific primers. Next-generation sequencing was employed to sequence the complete genomes of the identified positive samples. Subsequently, phylogenetic, gene homology, molecular evolutionary and genetic variation analyses were conducted.

**Result:**

In 2021, a total of 32,177 adult female mosquitoes were collected from 15 counties in Guizhou Province, China. The predominant mosquito species identified were *Culex tritaeniorhynchus*, *Armigeres subalbatus* and *Anopheles sinensis*. Among the collected mosquitoes, three positive cultures were obtained from *Cx. tritaeniorhynchus* pools, revealing the presence of Quang Binh virus (QBV) RNA sequences. Phylogenetic analysis indicated that the three Guizhou isolates, along with the prototype isolate from Vietnam, formed distinct branches. These branches were primarily closely related to other QBV isolates reported in China. Comparative analysis revealed a high degree of nucleotide and amino acid homology between the Guizhou isolates and both Vietnamese and other indigenous Chinese isolates. Additionally, nonsynonymous single-nucleotide variants (SNVs) were observed in these strains compared to the QBV prototype strain.

**Conclusion:**

This study represents the first report of QBV presences in *Cx. tritaeniorhynchus* mosquitoes in Guizhou Province, China. Phylogenetic tree analysis showed that the three Guizhou isolates were most closely related to the QBV genes found in China. In addition, the study of the genetic characteristics and variation of this virus provided a deeper understanding of QBV and enriched the baseline data of these insect-specific flaviviruses (ISFVs).

**Graphical Abstract:**

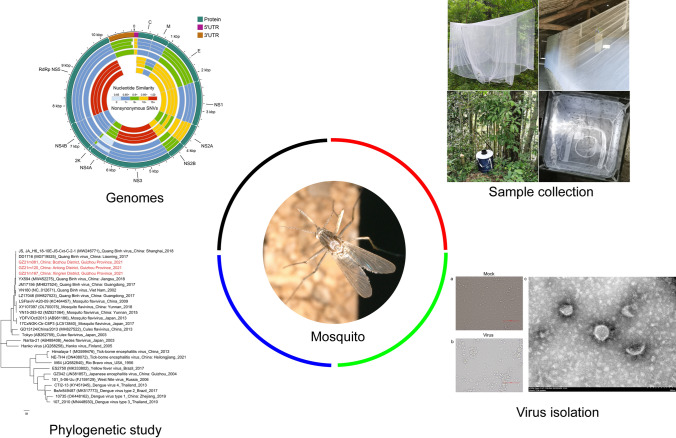

**Supplementary Information:**

The online version contains supplementary material available at 10.1186/s13071-023-05938-3.

## Background

Mosquitoes serve as significant vectors for various arboviruses, causing a significant burden on human health globally [1]. These arboviral diseases, including dengue, chikungunya, Zika virus disease and Japanese encephalitis, pose substantial public health concern [2]. Factors such as global warming, population movements, urbanization, increased international trade and modern agriculture and animal husbandry contribute to the rapid dissemination of mosquito-borne viruses, leading to the emergence and expansion of the diseases [3, 4]. Mosquitoes of *Aedes* spp. and *Culex* spp. are particularly important in transmission of flaviviruses (family *Flaviviridae*), alphaviruses (family *Togaviridae*), bunyaviruses (order *Bunyvirales*) and seadornaviruses (family *Reoviridae*). Flaviviruses have caused numerous emerging and re-emerging arboviral diseases [5, 6]. Notably, the introduction of West Nile virus from Africa to New York in 1999 led to its rapid dissemination and expansion [7, 8]. Similarly, Zika virus, initially discovered incidentally during yellow fever surveillance in Uganda in 1947, caused significant outbreaks in Americas between 2015 and 2016 [9]. Additionally, there was a severe dengue outbreak in Asia [10, 11]. Despite the prevalence of these viruses, effective vaccines are currently lacking for most members of *Flavivirus* genus, except for Japanese encephalitis virus and yellow fever virus, presenting a significant health challenge.

The flavivirus can be categorized based on their transmission vectors into three groups: tick-borne flaviviruses, mosquito-borne flaviviruses and flaviviruses with no known vectors [12, 13]. Additionally, there is a group of viruses known as insect-specific flaviviruses (ISFVs), which exclusively infect mosquitoes and have not been shown to infect humans or animals [14]. The ISFV was originally isolated from *Aedes aegypti* cells and classified as cell-fusing agent virus, which belongs to the family *Flaviviridae* [15]. Subsequently, Kamiti River virus, belonging to the same lineage of ISFVs, was isolated from *Aedes macintoshi* larvae and pupae in 1999 [16]. Besides cell-fusing agent virus and Kamiti River virus, several other ISFVs have been isolated and characterized, including *Culex* flavivirus [17] and *Aedes* flavivirus [18]. Since 1991, the presence of ISFVs has been documented worldwide [19, 20].

Guizhou Province, with its diverse natural environment, has been a hotspot for various mosquito-borne diseases and viruses, including Japanese encephalitis and Zika virus [21–23]. However, post mosquito surveillance efforts in Guizhou Province primarily focused on arboviruses, neglecting the presence and potential impact of insect-specific viruses (ISVs). Despite the perception of ISVs as nonpathogenic to humans, their ability to cross species barriers and cause disease should not be underestimated, as demonstrated by the identification of Liaoning virus [24].

Quang Binh virus (QBV) in the genus *Flavivirus* (family *Flaviviridae*) was initially isolated from *Culex tritaeniorhynchus* in Quang Binh City, Vietnam, in 2002 [25]. Subsequent studies identified QBV in various provinces and cities in China [19, 26, 27]. In our study, conducted in 2021, we isolated and identified QBV from mosquitoes collected in Guizhou Province. This a previously unreported flavivirus in Guizhou Province holds significance for understanding its origin, evolution, diversity and distribution. Furthermore, investigating QBV in mosquito viruses holds potential for utilizing ISFVs as biological control agents targeting vectors and medically significant viruses.

## Methods

### Survey area and mosquito collection

**Fig. 1:**
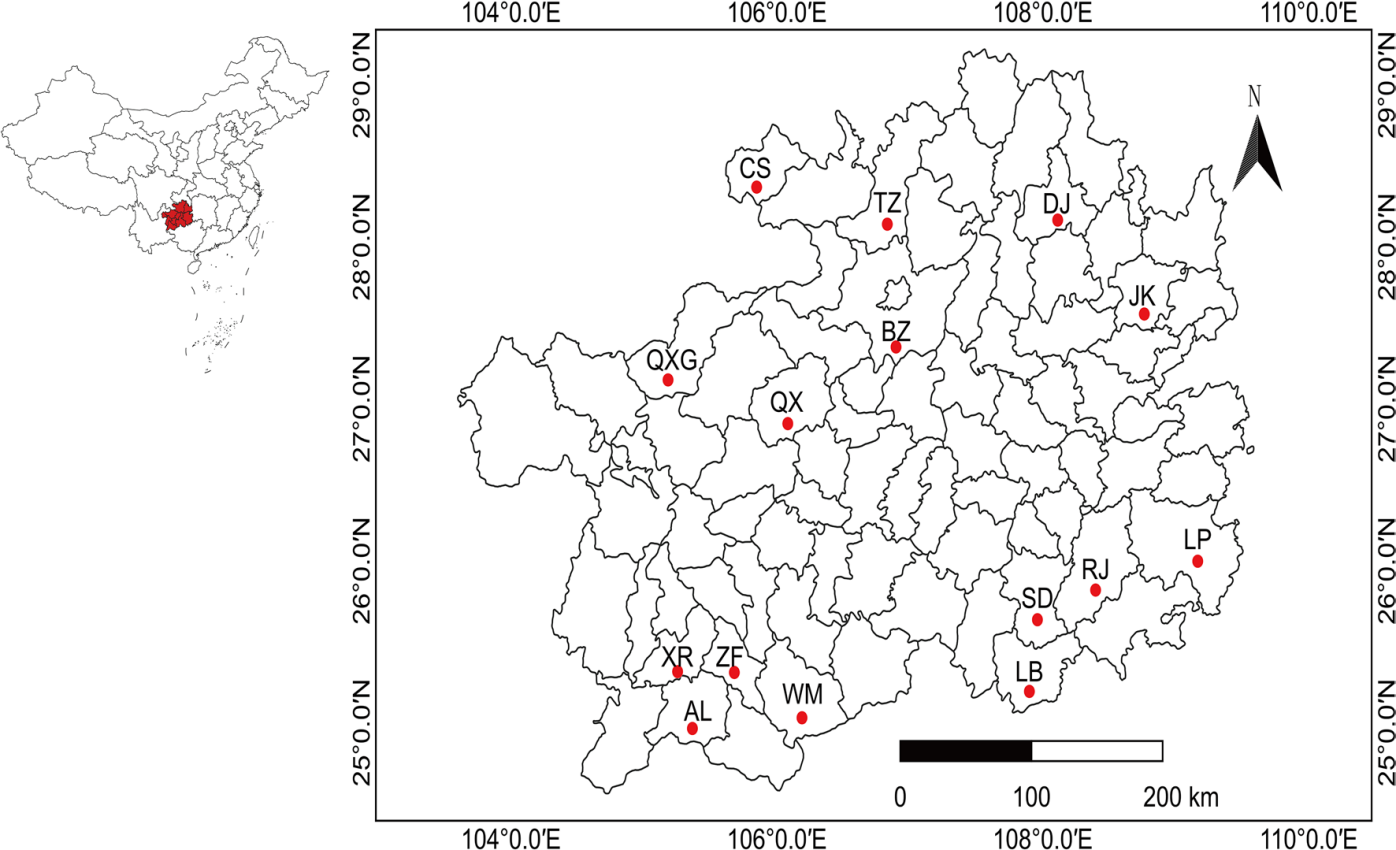
Map of mosquito collection sites in Guizhou Province, China. The acronym stands for counties and districts in Guizhou Province, China. The sampling sites are indicated by the red circle (BZ, Bozhou; CS, Chishui; TZ, Tongzi; DJ, Dejiang; JK, Jiangkou; XR, Xingren; ZF, Zhenfeng; AL, Anlong; WM, Wangmo; QXG, Qixingguan; QX, Qianxi; SD, Sandu; LB, Libo; LP, Liping; RJ, Rongjiang). National Earth System Science Data Center, National Science & Technology Infrastructure of China (http://www.geodata.cn)

Mosquitoes were collected from July to September 2021 from surveillance sites in 15 counties and districts in Guizhou Province, China (E 103°36′–109°35′, N 24°37′–29°13′) (Fig. 1), covering different types of mosquito habitats, including pig pens, cattle pens, field environments and other sites. Mosquitoes were collected using a light trap, a BG-Sentinel trap with BG lure and CO_2_ (Biogents, Germany) and a portable electroaspirator. Adult mosquitoes were frozen at − 20 °C for 30 min and placed on ice for morphological identification using a dichotomus key [28] and to remove male mosquitoes. Some species could not be clearly distinguished by morphological characteristics, and these were identified by a molecular biology method based on cytochrome C oxidase subunit I [29]. Female mosquitoes were then divided into pools of approximately 50–100 individuals each, based on species, date and location of capture. The pools were stored in liquid nitrogen tanks.

### Virus isolation

The virus was successfully isolated using the Vero cell culture method [30]. After thawing, the mosquitoes were washed three times with precooled and sterile PBS solution (Solarbio, Beijing, China). Each pool of mosquitoes was homogenized in 1 ml of RPMI-1640 medium (Gibco, Beijing, China) using a cryogrinder (Jingxin, Shanghai, China) with stainless steel beads. Samples were then centrifuged at 12,000×*g* for 20 min at 4 °C. The supernatants were filtered through a 0.22-μm Millipore filter (25 mm diameter) and inoculated into 12-well plates of C6/36 (*Aedes albopictus*) and BHK-21 (baby hamster kidney) cells for 1 h with 100 μl filtrates, with two wells for each sample. Next, 1 ml of media containing 2% serum was added, and the incubation was continued for 6–7 days. Two wells in each plate were used as negative controls. C6/36 cells were maintained in RPMI 1640 medium containing 2% fetal bovine serum (FBS; Gibco, New Zealand) at 28 °C with 5% CO_2_, and BHK-21 cells were maintained in minimal essential medium (Solarbio, Beijing, China) containing 2% FBS at 37 °C with 5% CO_2_. All media were supplemented with 1% penicillin-streptomycin (HyClone, Cytiva, USA). Cells were scored daily for cytopathic effects (CPEs), and cultures were harvested from the plates when > 75% of the monolayer was affected by CPE. Positive cultures were processed three times to obtain pure cultures. If no cytopathic effect was observed after three passages, the sample was considered negative.

### Virus identification and electron microscopy

Total RNA was extracted from cell-positive cultures using the QIAamp Viral RNA Kit (Qiagen, Hamburg, Germany), and total RNA was reverse transcribed using the cDNA Reverse Transcription Kit (Vazyme, Nanjing, China). For the detection of flaviviruses and alphaviruses, polymerase chain reaction was performed using previously published methods and primers [31, 32]. Two primers were designed to target the QBV NS5 gene for conventional PCR (forward primer 5′-GAG TAC GAA GCT CTG GGA TTT C-3′, reverse primer 5′-CTA GTA TGG AAG CGG TCG TTA TT-3′). Primer sequences were designed using the GenBank reference sequence (accession number MH827524) and PrimerQuest Tool software (https://sg.idtdna.com/PrimerQuest/Home/Index). PCR was performed using a 2 × SuperNova PCR Mix (GenStar, Beijing, China) PCR system (total reaction volume 50 μl), which contained 25 μl of 2 × SuperNova PCR Mix, 2.5 μl of each primer (10 μM), 18 μl of sterile water and 2.0 μl of cDNA sample. A thermal cycling procedure (Bio-rad, USA) was performed at 98 °C for 3 min, followed by 30 cycles at 98 °C for 15 s, 55 °C for 15 s and 72 °C for 30 s, with a final extension step of 5 min at 72 °C. The amplified products were analyzed by agarose gel electrophoresis and then purified and sequenced in both directions by Sangon Biotech (Shanghai, China). BLAST alignment was performed on the above sequences (http://www.ncbi.nlm.nih.gov/BLAST).

The positive virus culture supernatants from the cells were collected and centrifuged at 20,000 × *g* for 30 min at 4 °C (JIDI-17R, Guangzhou, China) to remove the debris. Twenty microliters of virus suspension was placed with a pipette gun and dropped on a copper grid with carbon film for 3–5 min, and then filter paper was used to absorb the excess liquid. Then, 2% phosphotungstic acid was dropped on the copper grid to stain for 1–2 min, filter paper was used to absorb excess liquid, and the grid was dried at room temperature. The copper grids were observed under transmission electron microscopy and photographed.

### Second-generation sequencing and data analysis

The virus supernatant was centrifuged at 12,000×*g* for 2 min to remove cells. Virus supernatants were treated with 200 U Benzonase (Millipore), Tubro DNase I (Thermo Fisher Scientific) and 0.1 mg/ml RNase A (Sangon Biotech) followed by heat inactivation of DNases at 65 °C for 10 min. Viral RNA was then extracted using the Qiagen MinElute Virus Spin Kit according to the manufacturer's instructions. RNA was quantified using an Equalbit RNA HS Assay Kit (Vazyme Biotech Co.,Ltd). The RNA virome library was constructed using a sequence independent amplification method [33]. The libraries were quality checked on an Agilent 4200 Bioanalyzer and sequenced on an Illumina Nova Seq 6000 platform with 2 × 150 bp paired-end reads.

Raw data were processed using Fast [34] by filtering low-quality reads and trimming adapters to obtain clean data. Contig assembly was performed using metaSPAdes [35] with default parameters, except “-k 21,33,55,77,99,127.” Contigs > 500 bp were retained for downstream analysis. Next, the retained contigs were queried against the NCBI NT database using MegaBLAST [36] with default parameters and a cutoff value of 10^–5^. According to the blast score, the virus species with the highest identity was selected as the final annotation for a query contig. First, the reference genome sequence was downloaded: Quang Binh virus (MH827524). Then, the contigs annotated to the same virus were mapped to the corresponding reference genome using MegaBLAST with “-dust no -word size 18.” The mapped contigs were then manually assembled using the Seqman application in the DNASTAR software package. All reads were remapped to the annotated viral contigs using BWA [37] for quantification. Virome sequencing and subsequent data processing were performed by Chengdu Life Baseline Technology Co., Ltd.

### 5′ RACE and cloning full length

5′ RACE was performed using the HiScript-TS 5′/3′ RACE kit (Vazyme, Nanjing, China) following the manufacturer's instructions. RNA was extracted from positive virus isolates, and the quality of RNA was assessed by gel electrophoresis or bioanalyzer. The primers used for 5′ RACE were as follows and designed based on known sequence information: TTT GTT TTC CCC TCG TAG ACC TGC ACG C (5′ specific). The PCR products were purified and cloned. Sanger sequencing was then performed to determine the unknown end of the target sequence.

### Sequence alignments and phylogenetic analysis

Multiple sequence alignments were performed with the gene sequences of three Guizhou viruses and relevant viral sequences using MAFFT version 7.490 [38]. The sequence of the reference strain QBV was downloaded from the GenBank database as well as the sequences of other flaviviruses, namely mosquito flavivirus, *Culex* flavivirus, *Aedes* flavivirus, yellow fever virus, Japanese encephalitis virus, West Nile virus and dengue virus types 1–4 (DENV 1–4), (Additional file 1: Table S1). Sequences were analyzed using BioEdit (www.mbio.ncsu.edu/BioEdit/bioedit.html) and the DNAStar (Lasergene) program package (https://www.dnastar.com/). To generate a heatmap, TBtools [39] was used (https://github.com/CJ-Chen/TBtools/releases). A phylogenetic tree was constructed with MEGA11 [40] using the neighbor-joining method and by building a maximum composite likelihood distance model with 1000 bootstrap replicates. Visualization was performed using FigTree (FigTree v1.4.4 visualization, http://tree.bio.ed.ac.uk/software/figtree/).

### Analysis of viral genomes

Gene annotation of three Guizhou virus strains was referenced to QBV (NC_012671). Briefly, we first downloaded two major protein sequences from QBV genomes as input and then used Prokka software [41] to acquire gene annotation with the following parameters: – Kingdom Viruses – norrna. Each virus genome was annotated with two CDS regions (polypeptide and truncated polypeptide), the regions upstream of the first CDS were defined as 5'UTR regions, and the regions downstream of the last CDS were defined as 3′UTR regions. For more detailed annotation, three Guizhou genome sequences were further aligned to the 15 mat peptide sequences of QBV genomes using BLASTN [42], and the best alignment entry for each mat peptide was obtained. Each region matched with mat peptide was extracted and used for open reading frame (ORF) prediction using Prodigal [43] software.

For sequence variant detection, the complete genomes of each Guizhou virus strain were aligned to QBV using ClustalW [44], and the bases of each Guizhou virus genome that did not align to QBV were extracted as single-nucleotide variants (SNVs) using custom-written Python scripts. These SNVs were annotated by ANNOVAR software [45], SNVs in the coding region were divided into synonymous SNVs and nonsynonymous SNVs, and the number of nonsynonymous SNVs in each mat-peptide region was counted. The similarity calculation between the Guizhou virus gene (including 5'UTR, 3'UTR and mat-peptide genes) and QBV gene was based on blastn alignment results. Circos plots were constructed using Circos software [46]. The circularity was represented from outside to inside as follows: tracks: 1, genomic structural regions; 2–5, nucleic acid sequence similarity (GZ21m081, GZ21m120, GZ21m167, mean); 6–9, number of nonsynonymous SNVs (GZ21m081, GZ21m120, GZ21m167, mean).

### Ribosomal frameshifting

With reference to the published literature, the reference genomes of Hanko virus (JQ268258), *Culex* flavivirus (AB262759.2) and Quang Binh virus (FJ644291) have been downloaded from NCBI. We manually searched for five match patterns that have been reported in insect flaviviruses (including GGAUUUC, GGAUUUU, GUUUUUU, UUUUUUU and UUUUUUC). Furthermore, RNA secondary structure prediction was performed 150 bp downstream of the matched site by using RNAfold software [47], and only the region downstream of the − 1 frameshift site with an RNA stem-loop structure was considered in the predicted results.

## Results

### Mosquito species field collection and composition

A total of 32,177 mosquitoes were collected from 15 counties of Guizhou Province, China. These mosquitoes were sampled from various locations, such as pig pens, cattle pens, trees, houses and other places. Among the collection sites, the largest portion of mosquitoes was obtained from cattle pens, accounting for 47.8% (15,379/32,177) of the total, followed by pig pens at 41.9% (13,495/32,177). These collected mosquitoes belonged to six species from four genera and two subfamilies, namely *Anopheles (An.)*, *Culex (Cx.)*, *Aedes (Ae.)* and *Armigeres (Ar.)*. Among the collected mosquitoes, *Culex* accounted for 53.5% (17,201/32,177), followed by *Armigeres* at 34.1% (10,962/32,177), *Anopheles* at 11.7% (3,750/32,177) and *Aedes* at 0.8% (264/32,177). The dominant species was *Cx. tritaeniorhynchus*, comprising 48.7% (15,661/32,177) of the collected mosquitoes, followed by *Armigeres subalbatus* (34.1%, 10,962/32,177) and *Anopheles sinensis* (11.7%, 3,750/32,177).

### Virus detection and characterization in vitro

In this study, mosquito samples were divided into 200 pools based on the location, time of collection and mosquito species, and virus isolation was performed on each pool by inoculating onto C6/36 and BHK-21 cells.

**Fig. 2:**
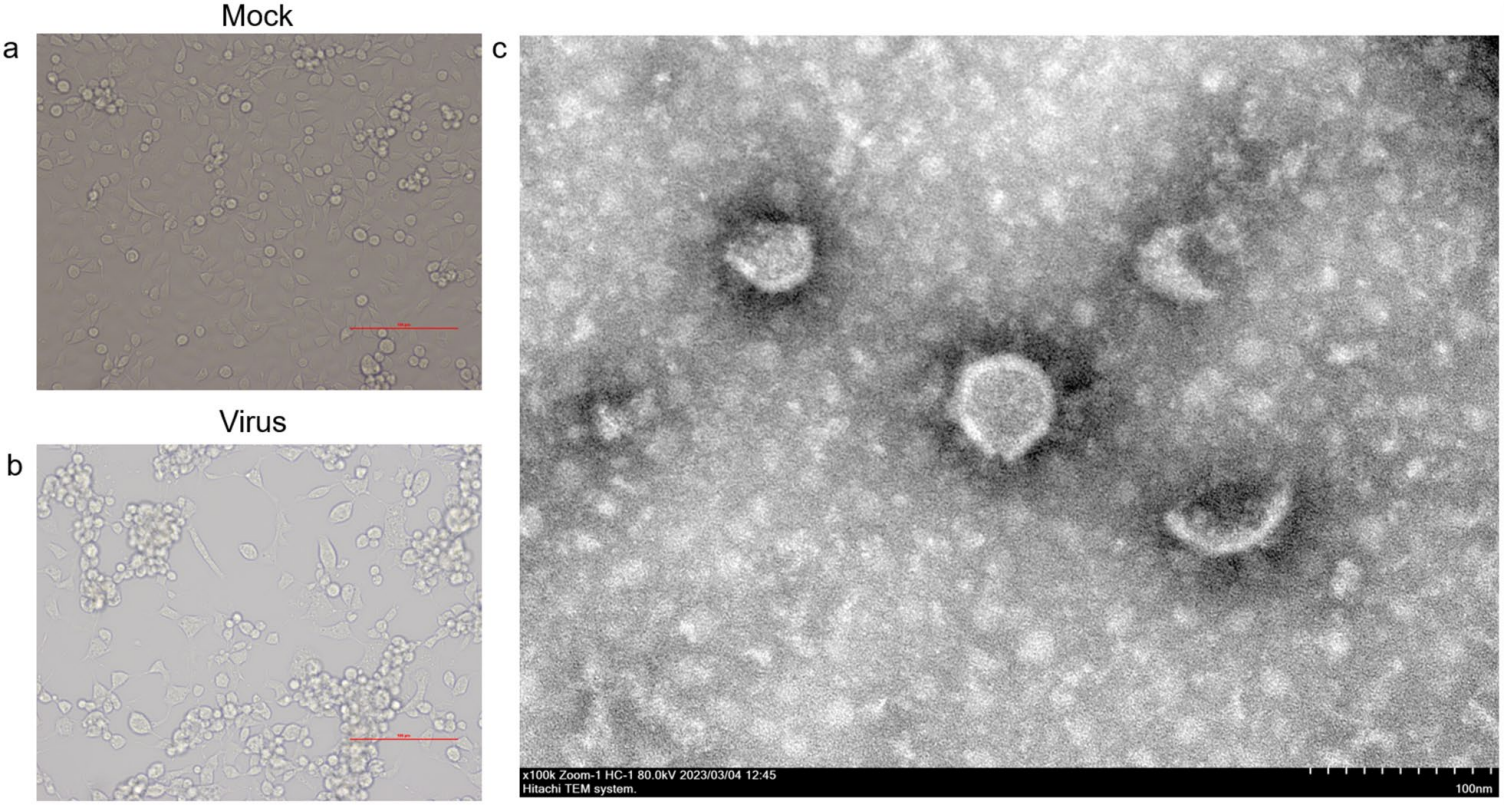
Isolates of Quang Binh virus were propagated in *Aedes albopictus* C6/6 cells. **a** Empty control cells (Mock) were used for infection testing. **b** C6/36 cells were infected with QBV to induce cytopathic effects (CPE). **c** QBV particles were visualized by transmission electron microscopy (TEM)

Positive cultures at passage 3 were screened for the presence of flaviviruses and alphaviruses using PCR. Three virus isolation cultures were found to be positive by the successful amplification of the partial QBV NS5 gene. An NCBI BLAST analysis of each sequence from each isolate showed a high degree of similarity to QBV (genus *Flavivirus*, family *Flaviviridae*). Of these, three virus isolates were from the *Cx. tritaeniorhynchus* pools, and they were collected from three areas (Table 1). In addition, these virus isolates caused a CPE in infected C6/36 cells, whereas no CPE was observed from inoculation on BHK-21 cells, and it appeared that the C6/36 cells had aggregated, detached and rounded compared to Mock cells (Fig. 2a, b). Electron microscopy showed that the viral particles were spherical with an envelope and approximately 40–60 nm in diameter (Fig. 2c). The virus particles were similar to the images that have been observed for members of the *Flaviviridae* family.

**Table 1:** Summary of viruses isolated from Guizhou in this study

Strains	Collection date	Host	Habitat	Geographic location
GZ21m081	07/2021	*Culex tritaeniorhynchus*	Pigpen	Bozhou District
GZ21m120	08/2021	*Culex tritaeniorhynchus*	Pigpen	Anlong District
GZ21m167	07/2021	*Culex tritaeniorhynchus*	Pigpen	Xingren District

### Complete genome sequencing

Next-generation sequencing was processed by filtering out low-quality reads and removing adapters. The sequencing results showed a total of 6,279,672 reads for GZ21m081, 4,999,228 reads for GZ21m120 and 8,267,166 reads for GZ21m167. In addition, the ratio of viral reads to clean reads was 66.0%, 90.1% and 64.7%, respectively. Reads below a minimum length of 30 nucleotides were trimmed, ensuring a minimum base quality of 99%. Subsequently, the trimmed reads were mapped to the reference sequence. The sequences of three Guizhou virus strains exhibited > 90% integrity and similarity to the reference sequence. The complete coding sequences of three Guizhou virus strains (GZ21m081, GZ21m120 and GZ21m167) exhibited the characteristic organization commonly found in flavivirus genomes. The complete genome sequencing of GZ21m081 revealed a total genome size of 10,831 nt, consisting of a 98-nt 5′-end noncoding region, 10,080-nt coding region and 653-nt 3′-end noncoding region. Similarly, the total genome size of GZ21m120 was 10,787 nt, with a 98-nt 5′-end noncoding region, 10,080-nt coding region and 609-nt 3′-end noncoding region. For GZ21m167, the total genome size was 10,678 nt, but the 5′-end noncoding region was not amplified. A 632-nt fragment was amplified using 5′ RACE, and a 10,829-nt viral genome was obtained via sequence splicing. The sequence consisted of a 117-nt 5′-end noncoding region, a 10,080-nt coding region and a 632-nt 3′-end noncoding region. BLAST analysis confirmed the similarity of the complete genome sequences in this study to other QBV isolates and references. The three genome sequences have been registered in the NCBI GenBank with the following sequence numbers: OQ139646, OQ139647 and OQ139648.

### Phylogenetic and homology analysis

**Fig. 3:**
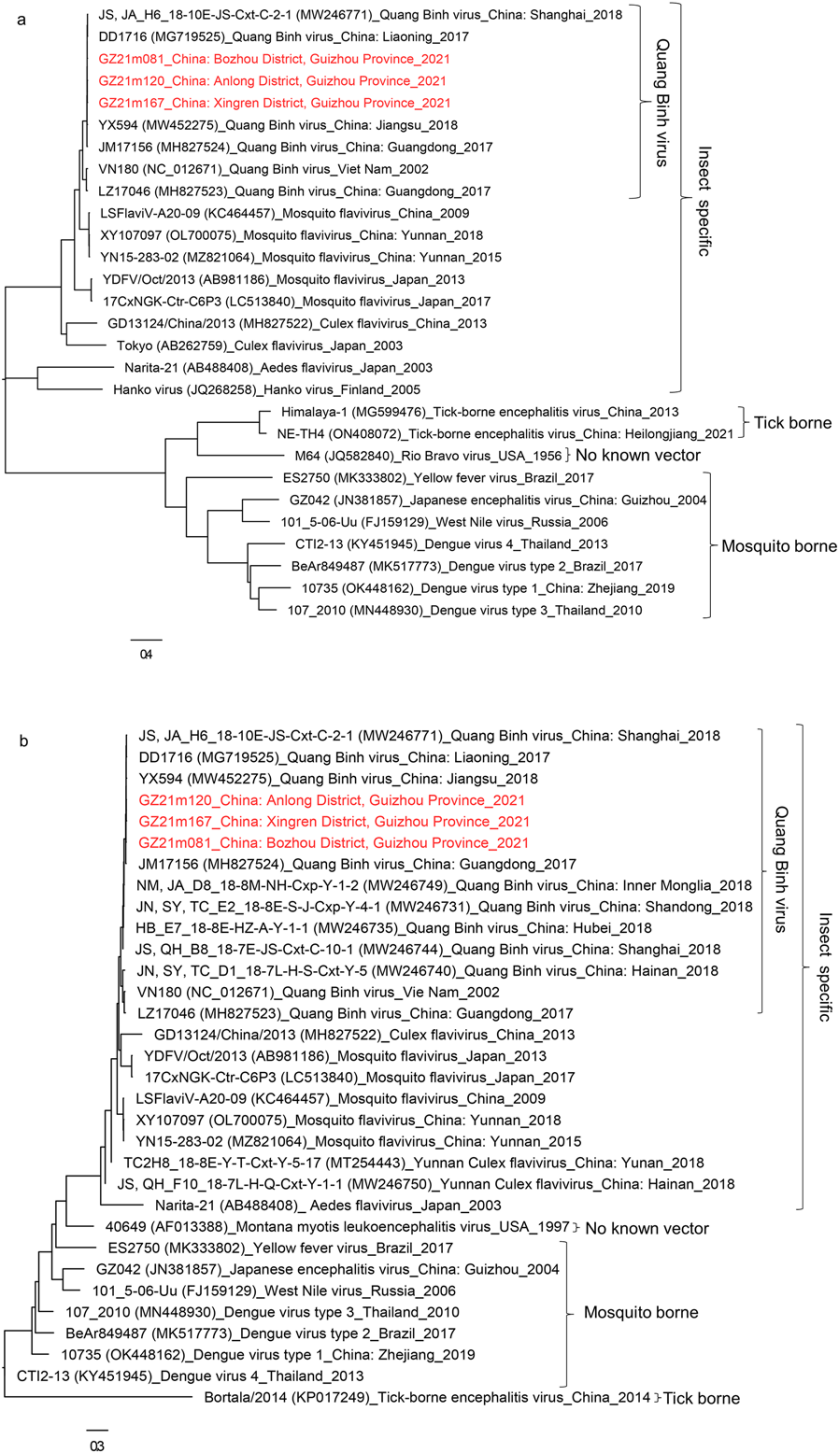
Phylogenetic analyses of the nucleotide sequences of flaviviruses. Phylogenetic trees constructed based on the complete genome sequences (**a**) and the NS5 gene (**b**). The evolutionary history was inferred using the neighbor-joining method. The optimal tree is shown. The percentage of replicate trees in which the associated taxa clustered together in the bootstrap test (1000 replicates) are shown next to the branches. The tree is drawn to scale, with branch lengths in the same units as those of the evolutionary distances used to infer the phylogenetic tree. The evolutionary distances were computed using the maximum composite likelihood method and are in units of the number of base substitutions per site. The rate variation among sites was modeled with a gamma distribution (shape parameter = 1). Evolutionary analyses were conducted in MEGA 11. Guizhou isolates identified in this study are labeled with red shading

The phylogenetic tree analysis based on the complete genome and the NS5 gene (Fig. 3) revealed the presence of four major groups of viruses within the *Flaviviridae* family: mosquito-borne flaviviruses, tick-borne flaviviruses, no-known vector flaviviruses and ISFVs. The analysis of the sequences (GZ21m081, GZ21m120 and GZ21m167) from Guizhou revealed that they belonged to the insect-specific flaviviruses and were grouped in the QBV clade. Interestingly, QBV exhibited two distinct branches in the clustering analysis. One branch comprised mainland Chinese strains from Shanghai, Liaoning, Jiangsu and Guangdong, while the other branch contained the first reported QBV strain from Vietnam (VN180) and a strain from Guangdong, China (MH827523). When compared to other members of *Flavivirus* genus, all Guizhou virus isolates formed a distinct group alongside QBV strains from China. The phylogenetic tree of the complete genome sequence (Fig. 3a) displayed a tree topology consistent with that obtained with the NS5 gene sequence (Fig. 3b).

**Fig. 4:**
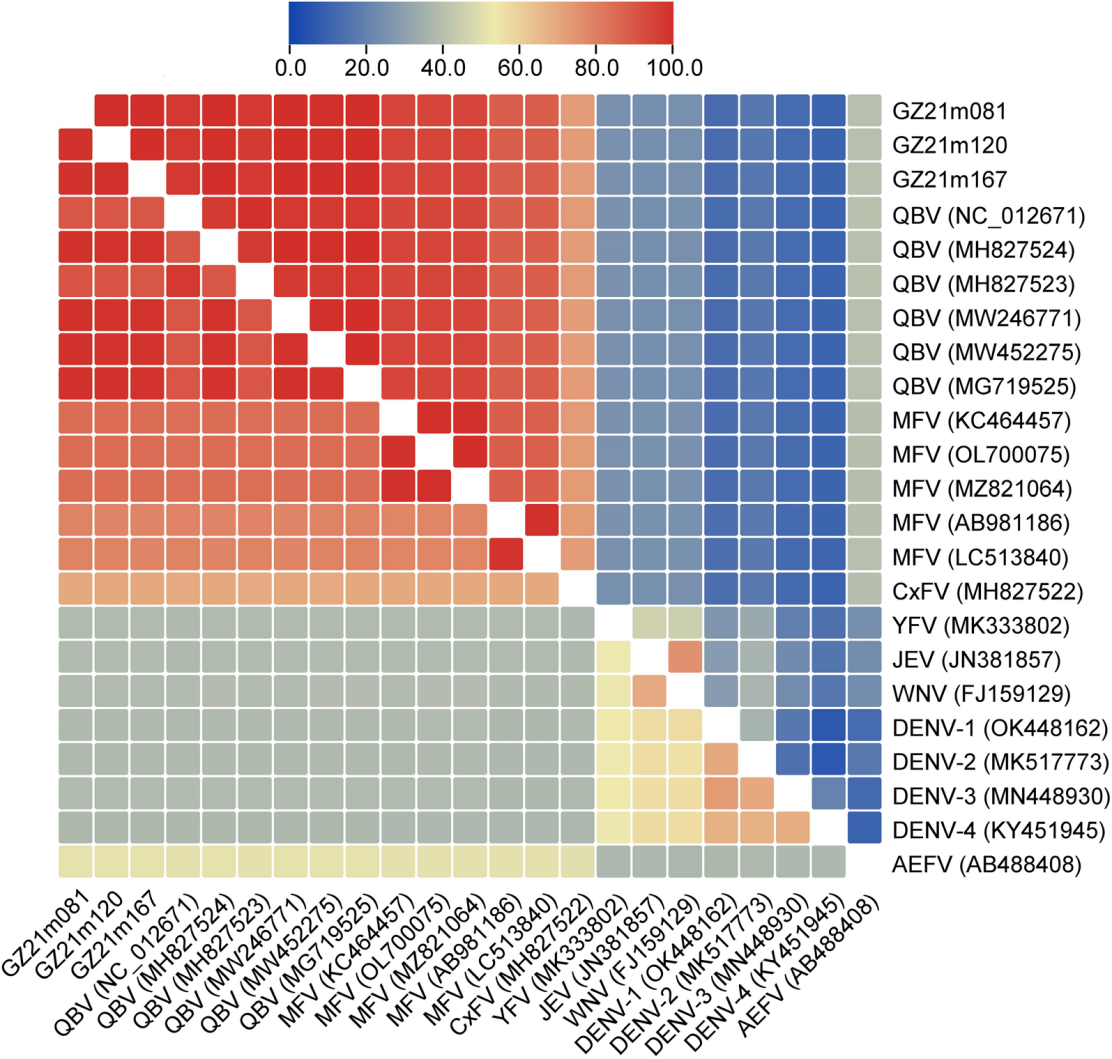
Heatmap of nucleotide and amino acid similarity in the flavivirus coding regions among QBV isolates in Guizhou and the reference strain and other flaviviruses. The similarity of amino acid sequences is shown in the upper triangle, and the similarity of nucleotide sequences is shown in the lower triangle. QBV, Quang Binh virus; MFV, mosquito flavivirus; CxFV, *Culex* flavivirus; YFV, yellow fever virus; JEV, Japanese encephalitis; WNV, West Nile virus; DENV (1–4), dengue virus (type 1–4); AEFV, *Aedes* flavivirus

The three QBV strains isolated from Guizhou Province were used for homologous comparison based on the complete coding region (10,080 nt) and deduced amino acids (3,359 aa). Heatmap analysis revealed that the three Guizhou strains were highly homologous to QBV at the nucleotide and amino acid levels (Fig. 4). Compared with the VN180 strain (NC_012671), the nucleotide and amino acid homologies of the three Guizhou virus strains were 90.0% and 97.2%, respectively. Furthermore, the three Guizhou virus sequences were highly homologous to JS, JA_H6_18-10E-JS-Cxt-C-2-1 (MW246771), DD1716 (MG719525), YX594 (MW452275) and JM17156 (MH827524) from China, with nucleotide sequence and amino acid identities ranging from 98.7 to 99.0% and 99.6 to 99.9%, respectively. Furthermore, the nucleotide and amino acid similarities with mosquito flaviviruses ranged from 78.5 to 84.4% and 87.7 to 94.4%, respectively, and with other mosquito-borne flaviviruses ranged from 37.4 to 69.2% and 10.3 to 72.4%, respectively.

### Comparative genomic analysis of QBV

**Fig. 5:**
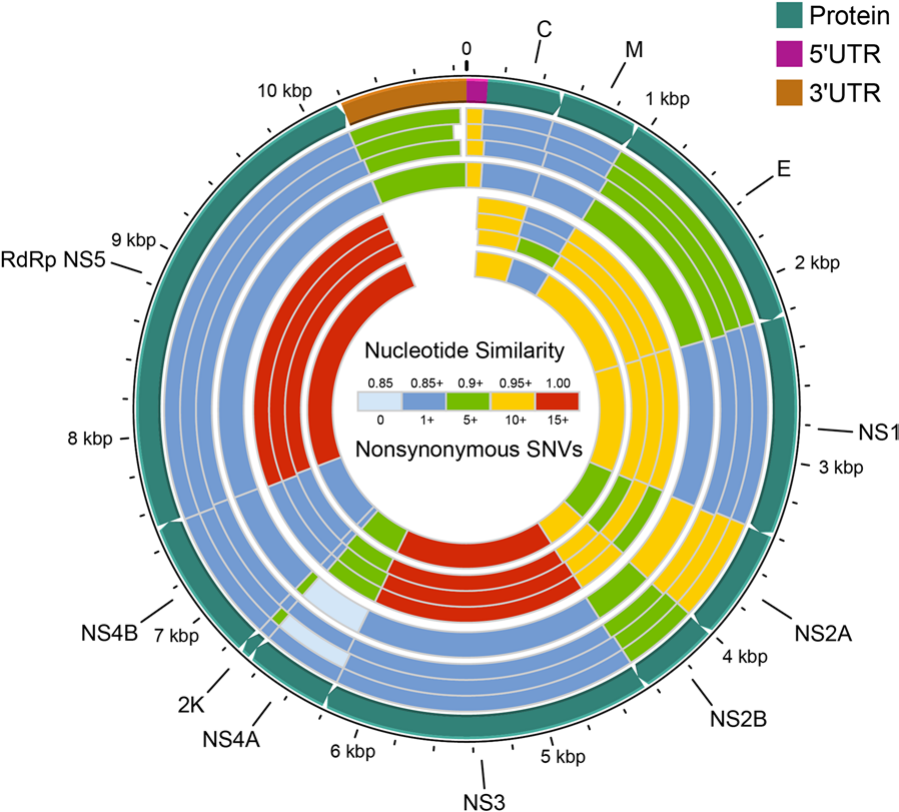
Comparative genomic analysis of three Guizhou virus genomes with Quang Binh virus. Circos plots from outer to inner represent: circle 1, genomic structural regions; circles 2–5, similarity of nucleic acid sequence to QBV (GZ21m081, GZ21m120, GZ21m167, mean); circles 6–9, number of nonsynonymous SNVs (GZ21m081, GZ21m120, GZ21m167, mean). Some overlapping genes are not shown in the circle diagram. C, capsid protein C; M, membrane glycoprotein M; E, envelope protein E; NS1, nonstructural protein NS1; NS2A, nonstructural protein NS2A; NS2B, nonstructural protein NS2B; NS3, nonstructural protein NS3; NS4A, nonstructural protein NS4A; 2 K, protein 2 K; NS4B, nonstructural protein NS4B; RdRp NS5, RNA-dependent RNA polymerase NS5

Circular maps, BLAST analysis and nonsynonymous SNV were used to illustrate three Guizhou virus genomes (Fig. 5). Nucleic acid similarity analysis showed that the E genes of the Guizhou viruses had the highest similarity to the reference genome (VN180, NC_012671), with an average similarity of 90.4%. The other structural protein genes exhibited average nucleotide sequence similarities ranging from 88.4 to 90.0%. Compared to the reference nonstructural protein genes (VN180), the NS2A genes of the Guizhou viruses showed the highest average nucleotide sequence similarity (95.9%), while the NS4A genes had the lowest (85.9%). The noncoding regions displayed high nucleotide sequence similarity to the reference genome, with average nucleotide sequence similarities of 95.6% for the flanking 5′ UTR and 94.6% for the 3′ UTR (Additional file 2: Table S2).

Compared to the reference sequences (VN180, NC_012671), the three Guizhou genomes exhibited 1050, 1049 and 1058 SNV sites, respectively. Among these SNVs, there were 104, 106 and 105 nonsynonymous SNVs, respectively (Table 2). The SNVs of the three Guizhou genomes in different genomic regions showed that the average number of structural protein nonsynonymous SNVs ranged from 1.6 in the protein pr to 13 in the anchored capsid protein C, while the AC + C and E regions displayed the greatest differences. In nonstructural proteins, the average number of nonsynonymous SNVs ranged from 1 in protein 2K to 17.6 in NS3, with NS3 and RNA-dependent RNA polymerase NS5 being the most abundant nonstructural proteins (Fig. 5, Additional file 2: Table S2).

**Table 2:** SNVs of Guizhou isolates with the corresponding reference strain VN180 (Quang Binh virus, NC_012671)

Region	Start	End	SNVs			Nonsynonymous SNVs		
			GZ21m081	GZ21m120	GZ21m167	GZ21m081	GZ21m120	GZ21m167
AC + C	113	520	44	41	43	13	13	13
PrM + M	521	946	49	47	52	3	3	5
E	947	2227	125	121	123	12	11	11
NS1	2228	3406	125	123	124	10	10	10
NS2A	3407	4012	25	26	23	9	11	9
NS2B	4013	4450	31	35	32	11	12	11
NS3	4451	6214	200	205	204	17	18	18
NS4A	6215	6679	64	67	65	8	9	9
2 K	6680	6748	7	6	7	1	1	1
NS4B	6749	7522	86	91	90	2	3	3
NS5	7523	10,189	294	287	295	18	15	15

*AC* + *C* anchored capsid protein C + capsid protein C, *PrM* + *M* membrane glycoprotein precursor M + membrane glycoprotein M,* E* envelope protein E, *NS1* nonstructural protein NS1, *NS2A* nonstructural protein NS2A, *NS2B* nonstructural protein NS2B, *NS3* nonstructural protein NS3, *NS4A* nonstructural protein NS4A, *2K* protein 2K, *NS4B* nonstructural protein NS4B, *NS5* RNA-dependent RNA polymerase NS5

In this study, nonsynonymous SNVs were analyzed separately for different ORFs. In terms of the number of nonsynonymous SNVs, the range of the number of nonsynonymous SNVs occurring in different ORFs was from 1 to 18. The highest number of nonsynonymous SNVs occurred in NS3 with an average of 17.7, followed by NS5 and AC + C with 16 and 13 nonsynonymous SNVs, respectively, and 2 K with the lowest number of 1 nonsynonymous SNV (Table 2, Additional file 2: Table S2).

**Fig. 6:**
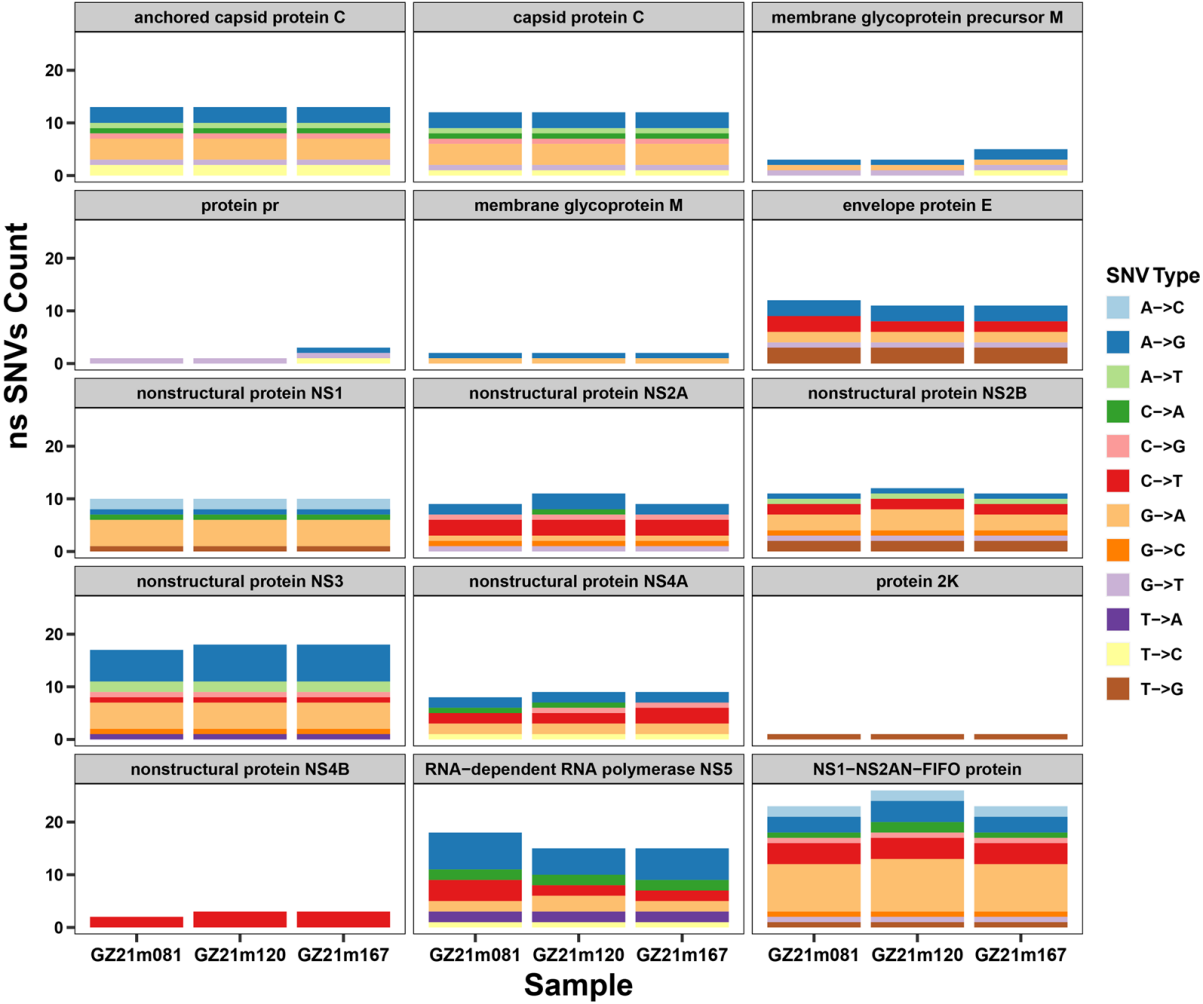
Distribution and number of nonsynonymous SNV variabilities among all Guizhou virus genomes. The variable nucleotide positions are based on the QBV prototype sequence (VN180, NC_012671). ns SNVs, nonsynonymous SNVs

Nonsynonymous SNV analysis across different ORFs revealed that mutations are present in all ORFs of the three Guizhou genomes. The highest number of mutations was observed in NS3, NS5 and AC + C (Fig. 6). The predominant mutation type in NS3 and NS5 was A to G, accounting for 35.3–38.9% in NS3 and 33.3–40.0% in NS5, respectively. In contrast, the mutation type in AC + C was G to A, with a maximum proportion of 30.8% (Additional file 3: Table S3).

### Ribosomal frameshifting

**Fig. 7:**
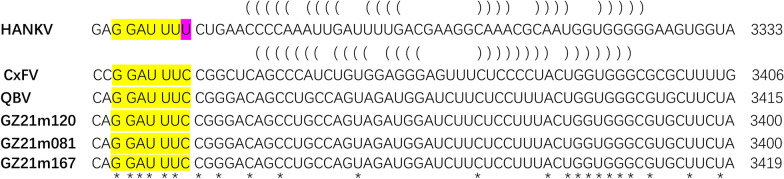
Predicted sites of ribosomal frameshifting in the genomes of Guizhou viruses and other insect flaviviruses. Yellow indicates the predicted − 1 frameshift site of the virus, pink indicates the mutated base at the frameshift site, brackets indicate the predicted RNA secondary structure, "*" indicates the conserved site, and the last number indicates the start position of the frameshift site. HANKV, Hanko virus (JQ268258); CxFV, *Culex* flavivirus (AB262759); QBV, Quang Binh virus (FJ644291)

We analyzed the presence of − 1 ribosomal frameshifting sites in the nucleotide sequences of three Guizhou virus genomes. Consistent with other ISFVs, the Guizhou genomes exhibited a conserved G_GAU_UUC slippery heptanucleotide (highlighted in orange) followed by a predicted occurrence in the NS2B region. However, HANKV had a different conserved sequence (G_GAU_UUU) in the NS2A-NS2B region. The frameshift site started at position 3333 (HANKV) to 3419 (GZ21m167). Additionally, a predicted RNA stem-loop structure was identified in the NS2B region (Fig. 7).

## Discussion

The *Flavivirus* genus within the family *Flaviviridae* consists of over 70 enveloped, positive-sense, single-stranded RNA viruses. These viruses are primarily transmitted by blood-sucking arthropods and are commonly found in vector mosquitoes [48]. Advances in sequencing technology and the expanded surveillance strategies have led to the discovery of novel flaviviruses specific to mosquito species. ISFVs such as Chaoyang virus, *Aedes* flavivirus, *Culex* flavivirus, QBV and Menghai flavivirus have been identified, with primary mosquito hosts (*Aedes*, *Culex* and *Anopheles* species) [6, 49, 50].

During our study, we found the predominant mosquito species in Guizhou province was *Cx. tritaeniorhynchus*, accounting for 48.7% of the collected samples, followed by *Ar. subalbatus* mosquitoes at 34.1%. The mosquito samples were primarily collected from cattle and pig pens. *Culex tritaeniorhynchus* vector, being the most common mosquito species in the province, plays a crucial role in the virus transmission, particularly as the principal vector of Japanese encephalitis [51]. Therefore, continuous monitoring of the main vector is necessary to investigate potential interactions between mosquito-borne viruses and other flaviviruses.

Our study identified the presence of ISF QBV in *Cx. tritaeniorhynchus* in the three regions of Guizhou province (Table 1). QBV from *Cx. tritaeniorhynchus* was first reported in Vietnam in 2002 [25] and subsequently detected in various provinces and cities in China but has not been reported in other countries [19, 26, 27]. We isolated only three QBV strains from 200 analyzed pools, resulting in a remarkably low virus isolation rate of 1.5% (3/200 pools). These findings suggest that QBV is not widely distributed throughout Guizhou Province, and further research is needed to determine its prevalence in nature. As reported in previous studies [52, 53], QBV has also been isolated from other mosquito species such as *An. sinensis*, *Ae. aegypti* and *Culex pipiens*, indicating its potential for multiple hosts. Therefore, it is crucial to focus on the vector species responsible for transmitting the virus among insects to better understand its transmission mode and potential mechanisms of spreads.

Phylogenetic analysis using MEGA software was performed to examine the genetic relationships of the three Guizhou virus sequences (GZ21m081, GZ21m120 and GZ21m167) within the QBV group. The analysis revealed that these three Guizhou virus sequences clustered with ISFVs QBV found in Chinese provinces (Fig. 3). Two main clades with different branch lengths were observed in the phylogenetic tree. One clade consisted mainly of strains/isolates from Chinese provinces such as Shanghai, Liaoning, Jiangsu and Guangdong, which were also present in our study. The other clade included the prototype isolated from Vietnam and other strains/isolates from Guangdong (MH827523) and Hainan (MW246740) provinces in China. These findings suggest the existence of different transmission chains of QBV in different countries and regions. Furthermore, the three Guizhou strains from our study exhibited high nucleotide and amino acid similarities with Chinese strains (MW246771, MG719525, MW452275 and MH827524), surpassing 98% similarity levels (Fig. 4). Additionally, our isolates showed a high level of nucleotide similarity (90.0%) with the Vietnamese prototype strain VN180 (Fig. 4).

Furthermore, the complete sequencing of the three Guizhou isolates allowed for a better understanding of their genetic relationships. The different regions of the sequences exhibited > 84% nucleotide similarity to the corresponding regions of the prototypical QBV isolate (Fig. 5). A species demarcation study [54] revealed a genetic similarity exceeding 84% among the three Guizhou strains, suggesting their close relationship with QBV within the flavivirus group. Therefore, these strains are classified as QBV because of their high degree of genetic similarity. However, it is important to note that QBV can be further divided into two distinct groups based on the phylogenetic tree and nucleotide similarity analysis. Currently, QBV has been reported only in Vietnam and certain provinces and cities in China, with limited documentation in other countries and regions. Given the limitations of this study, further surveillance in the future may provide valuable insights into the dynamics of QBV circulation.

Additionally, we conducted SNV analysis of the ORFs of the three Guizhou virus genomes and compared them with the corresponding regions of prototype QBV isolates. The analysis revealed that there were more synonymous mutations than nonsynonymous mutations, and the mutation sites and types were largely consistent among the three Guizhou virus genomes (Table 2, Fig. 6). Our analysis further revealed a notable degree of genetic consistency or similarity among the Guizhou QBV strains. Specifically, comparative genomic analysis identified a higher frequency of nonsynonymous SNVs in proteins encoded by NS3, NS5, AC + C, and E. The NS3 and NS5 proteins are essential for flavivirus replication [55], while the C protein plays roles in packaging viral genomic RNA and the formation of viral core [56]. Moreover, the E protein is crucial for virulence, stability and tissue tropism of flaviviruses [57]. Therefore, studying the effects of nonsynonymous SNVs on genetic diversity and protein expression may provide valuable insights for further research on mosquito-borne viruses.

Ribosomal frameshifting, specifically − 1 ribosomal frameshifting, is a well-defined process where ribosomes shift by one nucleotide and translate in a new reading frame [58]. Viruses utilize programmed − 1 ribosomal frameshifting to control gene expression and enhance the information content of their genomes [59]. ISFVs utilize programmed − 1 ribosomal frameshifting to express a new overlapping gene, *fifo*, in the NS2A-NS2B region [60, 61]. In the three Guizhou virus genomes, nonsynonymous SNVs were identified in NS2A, NS2B and NS1-NS2AN-FIFO. The predicted − 1 ribosomal frameshifting site contained a conserved G_GAU_UUC slippery heptanucleotide (Fig. 7), known to stimulate frameshifting [61]. These findings align with previous reports and support the existence of ribosomal frameshifting in the QBV genome [62], although further research is required to elucidate its mechanisms and functions.

Many aspects of the interaction among ISFVs, hosts, vectors and humans remain unclear [63, 64]. Surveillance efforts are crucial for systematically observing and tracking the prevalence, distribution and potential risks associated with both ISFVs and flavivirus arboviruses. We hypothesized that the co-infection of ISFVs and flavivirus arboviruses in mosquitoes may have a significant impact on human health, potentially influencing the transmission dynamics and pathogenesis of arboviruses in humans. Avian flu (influenza A viruses) provides an example supporting the hypothesis as avian flu viruses are typically harmless in birds but can cause severe disease in humans when they undergo genetic reassortment or mutation after infecting intermediate hosts, such as pigs [65]. Similarly, co-infections of ISFVs and flavivirus arboviruses in mosquitoes may facilitate genetic exchanges or modifications within the viral populations and lead to the emergence of new arbovirus variants with altered transmission patterns or increased pathogenicity in humans. Studying the interactions between ISFVs and flavivirus arboviruses in mosquitoes is crucial for understanding the potential consequences of co-infection. By investigating the genetic changes, altered transmission dynamics and increased disease severity that may result from these co-infections, we can develop better surveillance and control strategies to mitigate the risks associated with arboviral infections.

## Conclusions

Our study on wild mosquitoes carrying QBV demonstrates the One Health approach. It is the first report of QBV in *Cx. tritaeniorhynchus* in Guizhou Province. Phylogenetic analysis revealed that the three Guizhou isolates were most closely related to the QBV strains found in China. Furthermore, our comprehensive analysis of the complete genome and nucleotide variation in different genomic regions offers valuable insights into QBV evolution. This study significantly expands the baseline data of QBV genomes and provides a valuable resource for future research on molecular epidemiology, evolutionary studies and the development of molecular assays.

### Supplementary Information

**Additional file 1: Table. S1.** Background information on the Quang Binh virus analyzed in this study.

**Additional file 2: Table. S2.** Data of the circular diagram on nucleic acid similarity and nonsynonymous SNV analysis in this study.

**Additional file 3: Table. S3.** The results of nonsynonymous and synonymous SNV analysis in this study.

## Data Availability

The datasets used and analyzed during the current study are available from the corresponding author on reasonable request. FASTA files of genomes were deposited in NCBI GenBank and are available under the accession numbers OQ139646-OQ139648.

## Abbreviations

CPE: Cytopathic effects
ISFVs: Insect-specific flaviviruses
ISVs: Insect-specific viruses
ORF: Open reading frame
QBV: Quang Binh virus
RT‒PCR: Reverse transcriptase polymerase chain reaction
SNVs: Single-nucleotide variants
