# Supplementary material for: The identification and genetic characteristics of Quang Binh virus from field-captured Culex tritaeniorhynchus (Diptera: Culicidae) from Guizhou Province, China
Source: Parasit Vectors. 2023 Sep 7;16:318. doi: 10.1186/s13071-023-05938-3 (PMC10486134; doi:10.1186/s13071-023-05938-3)
Supplement: Supplementary file 1 — Additional file 1: Table. S1. Background information on the Quang Binh virus analyzed in this study. [file 13071_2023_5938_MOESM1_ESM.docx]

Table. S1. Background information on the Quang Binh virus analyzed in this study

| NO | Strain | Host | Collection date | Location | Accession No. | |
| --- | --- | --- | --- | --- | --- | --- |
|  |  |  |  |  | Complete genome | NS5 |
| 1 | VN180 | *Culex tritaeniorhynchus* | 2002 | Viet Nam | NC_012671 | NC_012671 |
| 2 | JM17156 | Mosquito | 2017 | China: Guangdong | MH827524 | MH827524 |
| 3 | LZ17046 | Mosquito | 2017 | China: Guangdong | MH827523 | MH827523 |
| 4 | JS,JA_H6_18-10E-JS-Cxt-C-2-1 | *Culex tritaeniorhynchus* | 2018 | China: Shanghai | MW246771 | MW246771 |
| 5 | YX594 | Mosquito | 2018 | China: Jiangsu | MW452275 | MW452275 |
| 6 | DD1716 | *Culex tritaeniorhynchus* | 2017 | China: Liaoning | MG719525 | MG719525 |
| 7 | LSFlaviV-A20-09 | *Culex tritaeniorhynchus* | 2009 | China | KC464457 | KC464457 |
| 8 | XY107097 | *Culex tritaeniorhynchus* | 2018 | China: Yunnan | OL700075 | OL700075 |
| 9 | YN15-283-02 | *Culicoides* sp. | 2015 | China: Yunnan | MZ821064 | MZ821064 |
| 10 | YDFV/Oct/2013 | *Culex* mosquitoes | 2013 | Japan | AB981186 | AB981186 |
| 11 | 17CxNGK-Ctr-C6P3 | *Culex tritaeniorhynchus* | 2017 | Japan | LC513840 | LC513840 |
| 12 | GD13124/China/2013 | Mosquito | 2013 | China | MH827522 | MH827522 |
| 13 | Tokyo | *Culex pipiens* | 2003 | Japan | AB262759 | - |
| 14 | ES2750 | Mosquito | 2017 | Brazil | MK333802 | MK333802 |
| 15 | GZ042 | *Armigeres* | 2004 | China: Guizhou | JN381857 | JN381857 |
| 16 | 101_5-06-Uu | Mosquito | 2006 | Russia | FJ159129 | FJ159129 |
| 17 | 10735 | *Aedes albopictus* | 2019 | China: Zhejiang | OK448162 | OK448162 |
| 18 | BeAr849487 | *Aedes aegypti* | 2017 | Brazil | MK517773 | MK517773 |
| 19 | 107_2010 | Mosquito | 2010 | Thailand | MN448930 | MN448930 |
| 20 | CTI2-13 | *Aedes aegypti* | 2013 | Thailand | KY451945 | KY451945 |
| 21 | Narita-21 | *Aedes albopictus* | 2003 | Japan | AB488408 | AB488408 |
| 22 | HB_E7_18-8E-HZ-A-Y-1-1 | *Anopheles sinensis* | 2018 | China: Hubei | - | MW246735 |
| 23 | JS,QH_B8_18-7E-JS-Cxt-C-10-1 | *Culex tritaeniorhynchus* | 2018 | China: Shanghai | - | MW246744 |
| 24 | NM,JA_D8_18-8M-NH-Cxp-Y-1- | *Culex pipien* | 2018 | China: Inner Mongolia | - | MW246749 |
| 25 | JN,SY,TC_E2_18-8E-S-J-Cxp-Y-4-1 | *Culex tritaeniorhynchus* | 2018 | China: Shandong | - | MW246731 |
| 26 | JN,SY,TC_D1_18-7L-H-S-Cxt-Y-5 | *Culex tritaeniorhynchus* | 2018 | China: Hainan | - | MW246740 |
| 27 | TC2H8_18-8E-Y-T-Cxt-Y-5-17 | *Culex tritaeniorhynchus* | 2018 | China: Yunan | - | MT254443 |
| 28 | JS,QH_F10_18-7L-H-Q-Cxt-Y-1-1 | *Culex tritaeniorhynchus* | 2018 | China: Hainan | - | MW246750 |
| 29 | M64 | No konwn | 1956 | USA | JQ582840 | - |
| 30 | 40649 | No konwn | 1997 | USA | - | AF013388 |
| 31 | Himalaya-1 | Marmota himalayana | 2013 | China | MG599476 | - |
| 32 | NE-TH4 | *Ixodes persulcatus* | 2021 | China: Heilongjiang | ON408072 | - |
| 33 | Bortala/2014 | *Ixodes persulcatus* | 2014 | China | - | KP017249 |
| 34 | Hanko virus | Mosquito | 2005 | Finland | JQ268258 | - |
